# Recommendations and Best Practices for Standardizing the Pre-Analytical Processing of Blood and Urine Samples in Metabolomics

**DOI:** 10.3390/metabo10060229

**Published:** 2020-06-03

**Authors:** Raúl González-Domínguez, Álvaro González-Domínguez, Ana Sayago, Ángeles Fernández-Recamales

**Affiliations:** 1 AgriFood Laboratory, Faculty of Experimental Sciences, University of Huelva, 21007 Huelva, Spain; ana.sayago@dqcm.uhu.es (A.S.); recamale@dqcm.uhu.es (Á.F.-R.); 2 International Campus of Excellence CeiA3, University of Huelva, 21007 Huelva, Spain; 3 Department of Pediatrics, Hospital Universitario Puerta del Mar, 11009 Cádiz, Spain; alhcadiz@gmail.com; 4 Institute of Research and Innovation in Biomedical Sciences of the Province of Cádiz (INiBICA), 11009 Cádiz, Spain

**Keywords:** metabolomics, pre-analytics, blood, urine, collection, processing, storage

## Abstract

Metabolomics can be significantly influenced by a range of pre-analytical factors, such as sample collection, pre-processing, aliquoting, transport, storage and thawing. This therefore shows the crucial need for standardizing the pre-analytical phase with the aim of minimizing the inter-sample variability driven by these technical issues, as well as for maintaining the metabolic integrity of biological samples to ensure that metabolomic profiles are a direct expression of the in vivo biochemical status. This review article provides an updated literature revision of the most important factors related to sample handling and pre-processing that may affect metabolomics results, particularly focusing on the most commonly investigated biofluids in metabolomics, namely blood plasma/serum and urine. Finally, we also provide some general recommendations and best practices aimed to standardize and accurately report all these pre-analytical aspects in metabolomics research.

## 1. Introduction

Metabolomics is nowadays one of the most powerful analytical approaches for studying the functional status of biological systems in clinical and biomedical research [1,2], toxicometabolomics and pharmacometabolomics [3], nutrition and food science [4,5], environmental metabolomics [6], and other research fields. As metabolites are the end-products of anabolic and catabolic pathways within living organisms, their comprehensive profiling may considerably help to holistically decipher the molecular mechanisms driving the final phenotype, which is in turn significantly influenced by numerous factors, including the genotype, pathological conditions and external factors (e.g., diet, pollution). The analysis of tissue samples and cells enables the in situ characterization of the localized and specific metabolic impairments in response to diseases or external exposures [7], but their accessibility is normally limited in clinical practice, unless using animal and cellular models, and it is often restricted to post-mortem samples. On the other hand, the use of biological fluids is generally preferred, not only due to their simpler and less invasive collection, but also because biofluids may provide deeper insights into the metabolome’s complexity at the systemic level, giving information about dynamic organ–organ network interactions, the presence of exogenous circulating metabolites, and the role of the host–microbiota interplay. In this vein, blood-derived samples (i.e., plasma and serum) and urine are the most frequently employed biological fluids in metabolomics, although a range of other biofluids have also been proposed, such as saliva, sweat, cerebrospinal fluid (brain-related disorders), bronchoalveolar lavage fluid (lung-related disorders), and many others [8].

**Figure 1:**
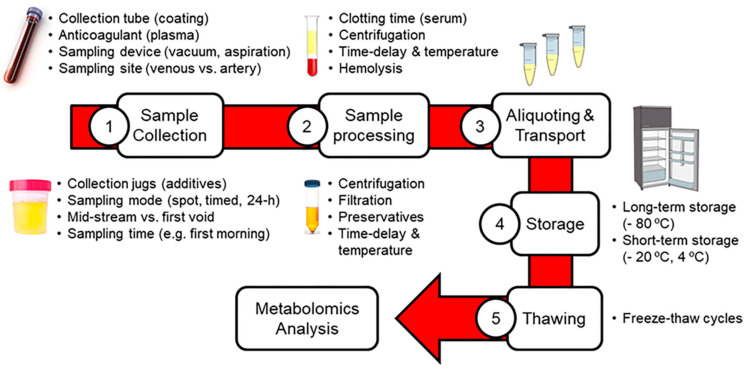
Summary of pre-analytical factors influencing blood and urinary metabolomics.

The great physico-chemical complexity and dynamism of the metabolome make the application of powerful analytical and computational approaches mandatory. There is a broad international consensus about the crucial role of proper sample preparation [9], the need for sensitive, specific and reproducible analytical platforms [10], and the utmost importance of data processing and statistical analysis [11] in the metabolomics workflow. However, other pre-analytical factors are sometimes overlooked in metabolomics, although they are critical as potential sources of variability. As summarized in Figure 1, the pre-analytical phase comprises various steps related to sample handling prior to analysis, including sample collection, pre-processing, aliquoting, transport, storage and thawing. Numerous studies have demonstrated that each one of these pre-analytical steps has a great impact on metabolite levels detected in biological samples, as recently reviewed for blood [12,13] and urine [14]. Although some authors have previously described that these pre-analytical metabolic alterations are often lower than the between-person variability [15,16,17], the need for standardizing the pre-analytical processing for ensuring reproducibility is nowadays beyond any doubt. This therefore shows the need for implementing standard operating procedures (SOPs) throughout the entire metabolomic pipeline, which is particularly relevant in large-scale multicenter studies and biobanking [18]. 

This review article is aimed to provide an updated literature revision of the most important pre-analytical factors influencing metabolomics results, focusing on plasma/serum and urine samples as the most commonly investigated biofluids. In this vein, it is noteworthy that most previous works published in this field have usually employed MS-based metabolomics platforms (e.g., LC-MS), and to a lesser extent NMR. On this basis, we provide some general recommendations and best practices with the aim of standardizing and accurately reporting all these pre-analytical aspects of metabolomics research.

## 2. Impact of Pre-Analytical Factors on Blood and Urinary Metabolomics

### 2.1. Sample Collection

The choice of the sample’s collection time is the first crucial factor to be considered in any study design, since metabolite levels are significantly influenced by a range of variables, such as the circadian rhythm, nutritional status, physical activity, and many other factors. Therefore, all samples throughout the entire study should be collected within the same time lapse (e.g., early morning) and under similar conditions (e.g., fasting) to minimize the impact of these factors on metabolomics results. Nonetheless, the simple collection of biological samples inherently alters the metabolomic profile, since additives contained in sampling devices and collection tubes may generate artefact signals. Therefore, all the materials employed for sample collection along the experimental setup should be purchased from the same manufacturer to avoid inter-sample variability due to chemicals released from the tubes and containers. However, the type of sampling devices and tubes used for sample collection is rarely described in most studies, which hinder the inter-comparability of results and the pooling of samples and/or data coming from different research centers. The collection of dried spot samples on filter papers is another alternative for simpler sampling, transportation and storage, but it is limited to small-sample volumes and its use significantly increases the complexity of subsequent analysis [19].

#### 2.1.1. Collection of Blood Samples

**Figure 2:**
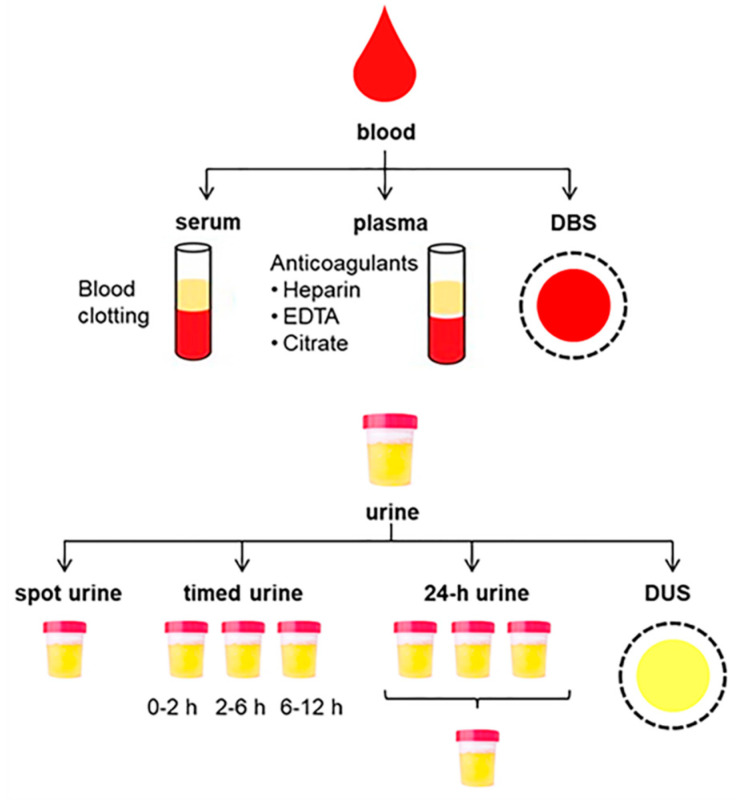
Summary of sampling alternatives for blood and urine collection.

Two different biofluids can be obtained from blood, namely serum and plasma, depending on the collection tube employed for allowing or blocking coagulation, respectively (Figure 2). To obtain serum, whole blood samples are left to clot and then centrifuged to separate the supernatant from the sedimented clot containing blood cells and clotting proteins, whereas anticoagulants must be used for obtaining plasma by centrifuging the non-clotted blood to separate cellular components from the liquid phase. Relatively small metabolomic differences have been reported between serum and plasma, but serum usually contains increased metabolite content and thus provides higher overall sensitivity. This could be in part explained by the volume displacement effect, which means that, during the coagulation of blood for producing serum, a fraction of proteins are removed, thus resulting in lower total volume and, consequently, higher concentration of solutes [20]. However, increased levels of some specific metabolites in serum compared with plasma could also be attributed to their release from blood cells during clotting (e.g., hypoxanthine, xanthine, amino acids) [21,22] or because of the activation and/or release of enzymes during this coagulation process (e.g., peptides derived from protease activity, lysophospholipids derived from phospholipases) [21,23]. Therefore, the clotting conditions for obtaining serum must be tightly controlled for minimizing enzymatic reactions and metabolomic alterations, paying special care to apply reproducible SOPs for ensuring low inter-sample variability. On the other hand, the advantages of plasma are the quicker and simpler processing, as well as better reproducibility compared to serum, because of the lack of this time-consuming and potentially variable clotting process [24].

Independently of the sample type, blood-derived biofluids can be contaminated with various exogenous interferences coming from the different components of the blood collection tubes, including rubber stoppers, surfactants and separator gels [25]. Among them, plastic polymers, plasticizers and slip agents, such as polyethylene glycol, phthalate esters or erucamide, have been proved to be major sources of contamination in mass spectrometry assays [26]. Furthermore, plastic consumables can also release metabolites endogenously present in biological samples (e.g., free fatty acids), thus hindering their analysis [27]. Tubes for serum collection are often coated with a polymeric film to activate blood clotting, but its use is not recommended in metabolomics because polymeric material residues may interfere with subsequent analyses [21]. In this sense, López-Bascón et al. found significant differences in the serum metabolic profile depending on the tube type used for collection (conventional or polymeric gel tube), with main changes being detected in amino acid metabolism (alanine, proline and threonine), glycerolipid metabolism (glycerol) and other important metabolites, such as monoglycerides (monopalmitin and monostearin), aconitic acid and lactic acid [28]. Similarly, the comparison of three different serum collection tubes (thrombin coated tubes, silicate coated tubes and conventional tubes) revealed an important influence of the coagulation process in metabolite levels, which was mainly reflected in higher content of the dipeptide phenylalanine–phenylalanine in silicate coated tubes [23]. On the other hand, plasma metabolomic profiles also show characteristic artefacts and noise signals depending on the anticoagulant employed for plasma processing. Cations from the anticoagulant agents (e.g., lithium, sodium, potassium) are known to contaminate plasma samples, thus significantly affecting MS-based metabolomics analysis by causing ion suppression and enhancement. For instance, it has been described that these cations can bind to negatively charged phospholipids, enhance their ionization and alter metabolomics/lipidomics profiles [29]. In this line, Yin et al. also reported that lithium ions from heparin may increase the ionization efficacy of phospholipids and triglycerides among other metabolites, aggravating matrix effects [30]. Sodium/potassium formate ion clusters, originated from the formate present in LC mobile phases and countercations from citrate and EDTA tubes, also play a major role in these ion suppression/enhancement processes, which may provoke serious matrix effects in the analysis of polar metabolites [21]. Moreover, anticoagulants may also impact various aspects related to sample preparation, such as the efficiency of the extraction and the derivatization processes; the latter occurs when GC-MS is employed, thus considerably influencing the analytical metabolomics coverage. The analysis of different plasma samples using an untargeted metabolomics approach demonstrated that the anticoagulant employed had a significant effect on levels of amino acids, carboxylic acids and sugar alcohols [31]. Particularly, authors found that EDTA is poorly suited to the analysis of polar metabolites, while the use of citrate tubes impedes analyzing citric acid and its derivatives. In another work, more metabolites could be detected in heparin plasma, both under positive and negative ESI-MS ionization, compared with samples collected in EDTA, citrate and oxalate tubes [32]. Khadka et al. reported that EDTA plasma presents a richer lipid profile (e.g., glycerophospholipids, sphingolipids, diacylglycerols, triacylglycerols, cholesteryl esters and acylcarnitines), and increased content of amino acids (e.g., aspartate, histidine and glutamine) than citrate ones [33]. However, a recent study also evidenced that EDTA tubes are not suitable for analyzing sarcosine, since blank EDTA tubes contained significant amounts of this metabolite [22].

Although the impact of the blood collection tube on metabolomics results has extensively been investigated as summarized above, other sampling-related aspects are also of great importance. For instance, it has been demonstrated that venous and artery bloods have different metabolic composition, so that the same extraction site must be used throughout the entire study for all the participants to avoid systematic bias [34]. The system employed for blood collection can also highly influence the final composition of blood-derived samples. Vacuum collection (e.g., Vacutainer^®^ tubes) is usually preferred since it represents a safe and standardized way of blood drawing, and its use ensures collection of constant blood volumes. However, aspiration systems (e.g., Monovettes^®^) enable control of the vacuum applied, thus reducing the risk of hemolysis (i.e., lysis of red blood cells). In any case, collection tubes must always be filled with the same volume of blood to ensure a reproducible concentration of additives and contaminants in all samples (e.g., anticoagulants in plasma, polymeric coating residues in serum, chemicals released from the tubes). Another study demonstrated that significant metabolomic differences can also be attributed to the alcohol wipes used for skin sterilization prior to blood drawing, with various surfactants, detergents, antimicrobials and stabilizers detected in blood samples [35].

#### 2.1.2. Collection of Urine Samples

Urine is also a common biofluid in metabolomics research because large volumes can be collected in a simple and non-invasive manner. Various sampling modes have been described for urinary bioanalysis, including spot sampling, timed sampling and collection of 24-h samples (Figure 2), which significantly influences the metabolic composition of these urine samples and must therefore be carefully selected depending on the study design and aims [36]. For random spot sampling, mid-stream samples (i.e., the intermediate portion of voided urine) have traditionally been preferred for urinalysis with the aim of minimizing contamination with bacteria, cells and particles from the genital mucosa and the urethra. However, a recent study reported that minor metabolomic differences are observed between first-void and mid-stream urine samples [37]. First morning collection immediately after the overnight rest, before breakfast and any physical activity, is usually recommended, since this urine is more concentrated than that collected at any other time during the day, thus being widely employed in nutrimetabolomics and exposure assessment [4]. By contrast, Liu et al. recommended the use of second morning urines for minimizing the effect of diet in urinary metabolomic profiles, thus simplifying the discovery of clinical biomarkers [38]. Another alternative is the collection of timed urine samples at different sampling points to investigate temporal metabolomic trends (e.g., kinetics of foods, nutrients or xenobiotics, metabolomic changes associated with the circadian rhythm). For this purpose, a control or blank urine sample is usually collected at baseline and then at various time periods (e.g., 0–2 h, 2–6 h, 6–12 h). All the urine samples collected within each interval are maintained under refrigeration and finally pooled. The third sampling method is the collection of all the urines throughout the entire day to be pooled in a single 24-h sample, which reduces the metabolomic variability observed at shorter sampling times, and gives an overall metabolic excretion picture. However, the collection of 24-h urine is cumbersome and error-prone, which usually requires the collection and proper storage of all urines at home by the study participants before sending to the research center. Therefore, although 24-h sampling could be preferable for minimizing variations in metabolite concentrations, single-spot urines are also widely employed in metabolomics because of their greater simplicity in clinical practice. In that latter case, the implementation of normalization strategies (e.g., creatinine, osmolality, specific gravity) becomes mandatory with the aim of correcting the inter-sample variability due to dilution factors [39,40].

Regardless of the sample type, bare polypropylene containers are normally employed for urine collection. Ji et al. recommended the use of surfactant additives to increase the solubility of lipophilic metabolites, which can suffer adsorptive losses due to their non-specific binding to the container walls [41]. However, surfactants have major repercussions on subsequent metabolomics analysis, especially when MS-based platforms are employed, so their use is not widespread. As previously mentioned for serum/plasma, special care must be paid to the selection of sampling jugs known to not release potential contaminants, such as plasticizers, into the sample. For timed and 24-h urine sampling, individual samples must be kept cool until subsequent pooling and storage. As explained below under Section 2.2.2, some preservatives can also be added to sampling containers to avoid bacterial growth and resulting metabolomic alterations.

#### 2.1.3. Dried Spot Sampling

The collection of dried blood spot (DBS), and to a lesser extent dried urine strip (DUS) samples, is another alternative for simpler sampling, transportation and storage of biological fluids in metabolomics research [19]. This methodology offers the opportunity for collecting biological samples away from the clinic, which can subsequently be sent to biobanks via postal service. To this end, a small drop of blood or urine (typically 25 μL) is spotted onto a filter paper card, and then left to dry before storage. Specifically, blood must be obtained by puncture on the fingertip or on the heel, the latter for children aged below 1 year, by using a lancet. This sampling procedure avoids the need for trained staff and the application of time-consuming centrifugation and other pre-processing steps, and shows increased stability without needing low temperatures for short term-storage. Accordingly, dried spot samples have widely been employed in numerous research fields, especially in studies dealing with limited amount of sample (e.g., neonatal screening of inborn errors of metabolism). However, some drawbacks of dried spot sampling include the release of background interferences from the filter paper, the difficulty of extracting metabolites strongly adsorbed into the paper, and lower sensitivity due to the reduced volume of sample.

### 2.2. Sample Pre-Processing

**Figure 3:**
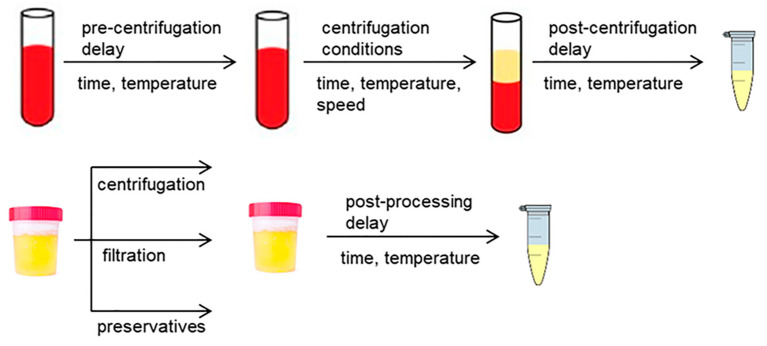
Summary of pre-processing factors influencing blood and urinary metabolomics.

Following collection and prior to storage, blood and urine matrices must normally be subjected to various pre-processing steps for obtaining the final sample, such as centrifugation, filtration, addition of preservatives and metabolic quenching (Figure 3). This pre-processing is of utmost importance to preserve the metabolic composition of biofluids with the aim of ensuring that metabolomics results obtained are direct and meaningful expressions of the in vivo biochemical status. However, it has repeatedly been reported that the pre-processing conditions and the time delay between sample collection and processing have a great impact on metabolite levels, which makes the implementation of standardized SOPs mandatory.

#### 2.2.1. Pre-Processing of Blood Samples

Blood processing conditions slightly differ depending on the need or not of the clotting step for producing serum or plasma, respectively. For obtaining serum, blood needs to clot at room temperature (RT) over a minimum period of 30 min prior to centrifugation, whereas plasma tubes can directly be processed once the blood is collected. This clotting time for serum may lead to considerable metabolic variability due to the release of compounds from activated platelets and because of enzymatic reactions, as previously described [21,24,35]. Conversely, plasma tubes can be put on ice until processing, thus minimizing undesired conversions and the lysis of red blood cells. In this context, numerous authors have investigated the influence of the time-delay and processing temperature between blood collection and centrifugation in metabolomics results. Overall, time delays until pre-processing at RT provoke important metabolomic alterations both in serum and plasma samples [42,43,44]. Particularly, Nishiumi et al. reported that some metabolite levels (e.g. pyruvic acid, hypoxanthine) change after just 15 min processing delay, these changes being more pronounced at higher time delays [45]. By contrast, the rate of these metabolic conversions is significantly slower when blood samples are refrigerated until their centrifugation. In this sense, various studies have demonstrated that only prolonged processing delays of several hours at cold temperature have a significant impact on serum/plasma metabolomic profiles [46,47,48,49], whereas others concluded that the majority of metabolites are stable for up to 24 h [50,51]. It is noteworthy that, although delays in blood processing affect both blood-derived biofluids, the impact on serum metabolites is normally higher [43] and occur after a shorter time delay [42]. Most of these studies have shown that the most important metabolic changes associated with delayed pre-processing and/or improper temperature storage until centrifugation are observed in energy-related metabolites (e.g., glucose, pyruvate, lactate), probably as a consequence of the anaerobic metabolism of erythrocytes [43,46,47,48]. Taking this into consideration, some authors have proposed different metabolite ratios measured in serum and plasma samples as markers of the pre-centrifugation conditions with the aim to determine the quality of blood samples for metabolomics analysis, such as the ascorbate to lactate ratio (i.e., Lacascore) [52], the lactate to glucose ratio [53], the ornithine to arginine ratio [54], or the lysophosphatidylcholines to phosphatidylcholines ratio [55]. 

The influence of the centrifugation conditions (i.e., centrifugation speed, brake force, temperature, time) in serum/plasma metabolomics has also been investigated in a few studies. Ammerlaan et al. reported that only the centrifugation temperature provokes minimal metabolic differences in serum and plasma samples centrifuged at 4 °C and 20 °C [56]. However, other authors did not find any effect of temperature, or other centrifugation conditions, on metabolite levels [47,48]. In another study, the plasma content of some metabolites (e.g., glutamine, sphingomyelins) was significantly affected by the centrifugal force applied, which could be attributed to substantial differences in platelet count [57]. Another crucial factor during blood pre-processing is the impact of hemolysis on metabolomics results. Common causes of blood hemolysis include strong aspiration during blood drawing, shaking of the blood collection tube, excessive centrifugal speed and inadequate pre-processing temperature, which may contaminate plasma and serum samples with hemoglobin and other erythrocyte components. This consequently leads to significant metabolic alterations associated with the release of specific metabolite classes from the intracellular space and the occurrence of metabolic reactions driven by erythrocyte-released enzymes [46,47,58,59]. The effect of hemolysis in serum and plasma metabolomes increases with the hemolysis level, so that the use of hemolyzed samples should be avoided in metabolomics studies to minimize unnecessary pre-analytical variability that might hinder data analysis and interpretation.

#### 2.2.2. Pre-Processing of Urine Samples

Bacterial metabolism and cells breaking upon freezing (e.g., bacteria, epithelial cells) may provoke significant alterations in the urinary metabolome. For these reasons, urine pre-processing prior to storage usually requires the application of a centrifugation or filtration step to remove cellular debris, bacteria and other materials in suspension. Saude and Sykes reported that filtration was the most effective strategy for preserving the urinary metabolome in samples stored for 4 weeks at RT and 4 °C [60]. In another study, the authors proposed that the combination of mild centrifugation with subsequent filtration is the best protocol to minimize urine contamination with compounds derived from cellular components [43]. However, they also highlighted that centrifugation at higher speeds provoked significant changes in metabolomic profiles, probably due to cell breakage. Accordingly, the protocol recommended by the European Consensus Expert Group Report for urine biobanking comprises these two steps prior to sample storage [61]. Nevertheless, it is well recognized that filtration may cause adsorptive losses of some metabolites, so that simple centrifugation is the most common procedure for urine pre-processing in practice. In this context, it has recently been described that the centrifugation temperature and brake speed can influence urinary metabolomics results [62]. Some authors proposed a protocol based on pre-centrifuging urine samples at 12,000 g for 20 min at 4 °C, which was validated in terms of microparticle counts, but did not take into consideration the possibility of cell breakage and consequent urine contamination, as previously reported [43]. Giskeødegård et al. found in another work that centrifuged and non-centrifuged urine samples show clear differences in metabolite levels, but this variability was much lower than that observed between subjects [37]. Therefore, they stated that the centrifugation step would not be a confounder of high influence in clinical studies.

The use of chemical preservatives (e.g., sodium azide, boric acid) is another alternative to minimize bacterial contamination of urine samples during collection and storage. The addition of boric acid has been demonstrated to prevent bacterial overgrowth and associated urinary changes in metabolite concentrations (e.g., acetate, creatine, amino acids, nucleosides) [63]. However, the storage of urine samples at 4 °C also inhibited bacterial contamination for at least 72 h without the need for any additive, and additionally avoided other alterations related to instability of some metabolites in solution. In another work, Smith et al. showed that boric acid readily forms adducts and complexes with hydroxyl and carboxylate groups, thus significantly impairing the detection of metabolites such as mannitol, citric acid and α-hydroxyisobutyric acid [64]. It was observed that the variation introduced by this complexation process was negligible in comparison with inter-individual variations, so borate preservation was proposed as a suitable method for ^1^H-NMR-based metabolomics, although special caution should be paid in the assignment of signals for metabolites having diol or adjacent hydroxyl and carboxylate groups. Similarly, Wang et al. also found that boric acid provokes metabolite changes in urine, and instead, recommended the use of thymol [65]. Nonetheless, they also demonstrated that urine metabolite concentrations remain stable at 4 °C for up to 48 h, thus showing that the use of additives is not mandatory. As an alternative, sodium azide is usually preferred as a preservative due to its lower impact on urinary metabolomic profiles. Various authors have reported that this bacteriostatic agent is very effective in inhibiting bacterial transformations, but its addition inherently alters the chemical urinary composition by introducing artefact signals and inducing changes in the pH and ionic strength [43,60,66]. Overall, all these previous works suggest that the beneficial effect of adding chemical preservatives for quenching bacterial metabolism is comparable to that obtained by keeping samples at cold temperatures until storage and the pre-processing of urine samples by means of mild centrifugation and/or filtration. Accordingly, the general recommendation of the European Consensus Expert Group is to avoid the addition of preservatives to urine samples [61]. However, their use is still highly advised in particular cases where urine cannot be immediately pre-processed and stored at cold temperatures, such as for instance the collection of urine samples from animal models in metabolic cages.

#### 2.2.3. Metabolic Quenching

Metabolic quenching is crucial to inactivate enzymatic reactions after sample collection with the aim of obtaining a precise picture of the metabolome at the time of sampling. This procedure is usually mandatory for tissue and cellular metabolomics, but it is generally omitted when analyzing blood and urine samples. This is mainly due to the relatively high metabolic integrity of these biofluids that, after their pre-processing and before long-term storage, can be stored either at RT for a few hours [46,48,67,68] or at 4 °C for up to 24–48 h [50,67,68,69] without suffering significant alterations in most metabolite levels. Nevertheless, it is nowadays well recognized that residual enzymatic reactions and chemical transformations induced by air oxidation and light exposure may cause some metabolic alterations during sample post-processing, such as the loss of easily oxidizable species [70], the conversion of labile metabolites (e.g., energy-related metabolites, nucleotides) [71,72], and the hydrolysis of lipids [73], among other processes. To prevent these metabolic transformations, snap-freezing in liquid nitrogen is the most commonly employed protocol for quenching, especially for metabolites with a short half-life. By contrast, metabolic quenching with dry ice is not recommended because carbon dioxide solubilization leads to non-reproducible pH changes in the sample, which may have repercussions on subsequent metabolomics analysis [74].

### 2.3. Aliquoting, Transport and Storage of Samples

The aliquoting of biological fluids must be performed at the moment of sampling, and before freezing the samples for their shipment and/or storage, with the aim of avoiding freeze-thaw cycles during subsequent analysis. The number of aliquots to prepare will depend on the study design and the number of analytical tests to be performed in these samples (e.g., application of complementary platforms for accomplishing a comprehensive metabolomics study) [75], whereas their volume should be sufficient for fulfilling the specific analytical requirements and allow for precise pipetting. As previously stated in Section 2.1, all aliquots should be collected in tubes from the same manufacturer to minimize systematic bias associated with chemicals released from the plastic ware. For samples frozen before aliquoting (e.g., home sampling), it is recommended to thaw samples on ice and rapidly prepare smaller aliquots and refreeze to minimize additional analytical variations. Alternatively, Zhang et al. have demonstrated that the use of frozen-sample aliquotter technologies allows extraction of homogenous aliquots suitable for metabolomics analysis without the need for thawing the original sample [76].

The transport of biological samples from the site of collection to the biobanks may significantly impair the quality of samples and consequently affect metabolomics results. This is particularly relevant in home-sampling studies, where samples are collected and shipped by the participants without the advice of experts. Ideally, biological samples should be pre-processed, aliquoted and frozen in the collection site, and then transported at cold temperature to avoid freeze-thaw cycles. In this vein, Breier et al. explained that blood samples must immediately be centrifuged to obtain serum or plasma prior to shipment, since various metabolites (e.g., amino acids, biogenic amines) become unstable within 3 h when non-centrifuged blood tubes are maintained on cool packs [51]. Another study demonstrated that plasma thawing during transportation provokes numerous alterations in levels of amino acids, fatty acids, glycerol metabolites and nucleotides as a result of protein and cell degradation processes, and increased phospholipase activity [77]. Similarly, Morello et al. also found significant metabolomic differences between urine samples collected at the hospital and those collected by patients at home and sent to the laboratories by post, which were mainly associated with increased bacterial contamination in samples collected outside of the clinical setting [78]. Nonetheless, the authors emphasized that samples collected at home still retain valuable physiological information and can be used in metabolomics. In this line, a recent study has also reported that urine sampling at home and storage in refrigerators until sending to the research center is acceptable for the quantification of biomarkers related to habitual dietary exposure [79].

Finally, sample aliquots must be stored at the lowest temperature as possible to ensure their stability. Overall, many studies have previously demonstrated that long-term storage at −80 °C does not considerably influence the metabolic composition of blood [47,48,80] and urine [66,81,82] samples. However, other authors found that longer storage for more than five years can lead to altered plasma levels of lipids, amino acids and hexoses [83,84,85]. In this regard, it should also be noted that biological samples must sometimes be stored at higher temperatures until their shipment to biobanks because of the often unavailability of ultra-freezers in many clinical settings. Thus, numerous studies have been conducted during the last years to evaluate the stability of urine and blood-derived biofluids under different storage conditions. For urine, it has been reported that freezing at −20 °C for up to 6 months [66,86,87], and storage in the fridge (4 °C) or using cooling packs (10 °C) for 24–72 h [63,81,86] allows preservation of the urinary metabolic profile. Nonetheless, small urinary alterations can been detected during the first days of storage, even at −80 °C, so that it is recommended to freeze urine samples for a minimum period of one week prior to conducting metabolomics analysis with the aim of ensuring a consistent evolution of urinary components in all samples across the study cohort [60]. More recently, Živković Semren et al. reported that these time-dependent changes are especially pronounced for urinary volatile metabolites unless samples are subjected to deep-freezing [88]. On the other hand, the high concentration of enzymes in blood makes serum and plasma samples more susceptible to metabolic alterations under sup-optimal storage conditions. Short-term storage of serum and plasma at 4 °C is only possible for a few hours before the first symptoms of enzymatic-driven metabolic conversions appear [46,55,58,84], whereas other authors have described that these samples can be maintained at −20 °C for up to one week before biobanking in ultra-freezers [80,89]. However, Moriya et al. reported that ensuring the stability of some labile metabolites is only possible at −80 °C, since the storage of blood samples above this temperature provoked significant alterations in lipids due to the action of lipases, drastic reductions in cysteine as a consequence of oxidation processes, as well as the accumulation of N^6^-methyladenosine and uracil reflecting RNA degradation [67].

### 2.4. Thawing of Samples

The availability of biological samples is a common problem in biobanking, especially when a proper aliquoting has not been performed. This inevitably forces one to accomplish repetitive freeze-thaw cycles for preserving valuable biological material for future investigations. To this end, stepwise thawing at 4 °C and immediate refreezing is mandatory to guarantee the metabolic stability of samples. Furthermore, all samples analyzed within an experimental design must have suffered the same number of freeze-thaw cycles to minimize inter-sample variability. However, it should be noted that this thawing process can lead to important metabolic perturbations that might reduce the validity of resulting metabolomics results. Many studies have demonstrated that plasma and serum samples subjected to one or two freeze-thaw cycles are not significantly altered from a metabolomics point of view [15,16,51,80]. Meanwhile, other authors have reported that a maximum of 3–4 freeze-thaw cycles is recommended to ensure the stability of the lipid profile in plasma and serum, since further thawing may induce changes in the levels of polyunsaturated fatty acids [90], and other high abundant lipids such as diglycerides, triglycerides, phospholipids, cholesterol derivatives and others [89]. Similarly, only repeated thawing has been demonstrated to have a deep impact in other metabolite classes detected in blood samples. In this sense, Anton et al. found slight increases in serum amino acids after four freeze-thaw cycles, which might indicate protein degradation processes [55]. Reduced L-carnitine was the only change detected in plasma after two and four thawing steps [58]. In another work, Fliniaux et al. observed a visible impact of 5 or 10 freeze-thaw cycles on the NMR metabolic profile of serum samples, with decreased choline, glycerol, methanol, ethanol and proline content [91]. In this line, more than three consecutive freeze-thaw cycles provoked significant alterations in plasma levels of lipids, choline-containing compounds, amino acids and energy-related metabolites [80]. Interestingly, authors found that the effect of thawing was strongly sample-dependent, with a larger impact on lipid-rich samples. On the other hand, urine seems to be more stable upon repetitive thawing compared with blood samples. Gika et al. showed that LC/MS-based urinary metabolomic profiles were not affected by the thawing of samples for up to nine freeze-thaw cycles [86], whereas in another study significant alterations were only observed after thawing urine samples twice a week over four consecutive weeks [60]. However, the effect of thawing is much more pronounced when considering urinary volatiles, which may be impacted after two freeze-thaw cycles [88].

## 3. General Recommendations for Blood and Urine Pre-Processing

Taking into consideration the huge impact of the pre-analytical phase on blood and urinary metabolomics, the collection and pre-processing of these biological fluids must be performed by using reproducible SOPs for minimizing inter-sample variability and ensuring that metabolomics results faithfully represent the in vivo biochemical status. We also recommend accurately reporting all these pre-analytical conditions for facilitating the pooling of samples and/or data coming from different research centers or biobanks. On the basis of the literature previously discussed in this review article, we provide here some general recommendations and best practices for standardizing these pre-analytical factors in metabolomics research, as shown in Table 1.

In summary, serum and plasma samples can indistinguishably be employed for metabolomics research, serum usually providing higher sensitivity, whereas better reproducibility can be achieved with plasma. The use of polymeric-coated collection tubes is not recommended for obtaining serum since they may introduce artefact signals [21,28]. On the other hand, the choice of the best anticoagulant for plasma collection is still under debate, although numerous studies have demonstrated that heparin significantly improves the metabolomics coverage [21,32], whereas the use of citrate must be avoided because it hampers the extraction of lipids and makes the determination citric acid and derivatives impossible [21,29,31]. Regarding urine, collection of spot, timed and 24-h samples provide complementary performances depending on the study aims [36]. For spot urine sampling, mid-stream collection of first morning samples is normally preferred, where the application of normalization strategies is necessary for correcting inter-sample variabilities associated with dilution factors [39]. Alternatively, the collection of timed and 24-h samples requires maintaining individual urine samples under refrigeration until pooling to preserve their stability. The subsequent pre-processing of biological fluids must be performed by using reproducible procedures, minimizing the time-delay and the exposure to improper temperatures between collection and further processing. After incubation for 30 min at RT for clotting (only for serum), blood collection tubes must be centrifuged according to the manufacturer’s instructions to separate the serum/plasma samples from the cellular fraction (e.g., 2000 g for 10 min at 4 °C). If immediate processing is unaffordable in the practice, prolonged delays must be avoided to minimize compositional alterations (< 30 min at RT, 1–2 h at 4 °C) [43,46]. Urine also requires the application of a mild centrifugation step to remove bacteria, cells and other materials in suspension prior to storage [43]. However, the addition of preservatives should be avoided unless urine samples cannot be immediately pre-processed and stored at cold temperatures (e.g., collection in metabolic cages) [61]. These pre-processed samples should immediately be aliquoted and frozen at −80 °C to preserve their metabolic integrity. The number and volume of aliquots to be prepared will depend on the study aims, and these should be not subjected to more than 2–3 freeze-thaw cycles [51,79]. With regards to the shipment of samples, if needed, it is generally recommended to carry out the sample pre-processing, aliquoting and freezing in the collection site whenever possible, and then transport them at cold temperature to avoid thawing. Long-term storage of biological samples requires the use of ultra-freezers (−80 °C), which maintain their metabolic integrity for up to 5 years [47,79,80,81]. If ultra-freezers are not available in the collection site, samples can be stored for shorter periods at 4 °C (1–2 h for blood, 24–48 h for urine) or at −20 °C (up to 1 week for blood, up to 6 months for urine) until their shipment to biobanks [79,83,85,88].

## 4. Conclusions

The pre-analytical phase has a great impact on metabolomics results, which makes the implementation of standardized procedures mandatory throughout the entire workflow, including sample collection, pre-processing, aliquoting, transport, storage and thawing before analysis. On the basis of a comprehensive literature revision, we have provided here some general recommendations for standardizing and accurately reporting all these pre-analytical steps in metabolomics studies. The first crucial need is the application of reproducible SOPs for minimizing the inter-sample variability driven by these pre-analytical factors. In general, it is preferable to minimize time delays between sample collection, pre-processing, and storage. Temperature during pre-processing and storage also influences metabolite levels in a great extent, and the number of freeze-thaw cycles must be kept to a minimum. Furthermore, the use of additives (e.g. preservatives) should be avoided whenever possible for maintaining the metabolic integrity of biological samples. However, there is still great debate concerning other important pre-analytical issues, such as the choice of the anticoagulant for obtaining plasma, the need of a quenching protocol for biofluids, or the impact of centrifugation conditions.

## Figures and Tables

**Table 1:** Recommendations for Standardizing the Pre-Analytical Phase in Blood and Urinary Metabolomics.

Pre-Analytical Factors		Blood	Urine
sample collection	sampling time	All the samples must be collected within the same time lapse, under similar fasting status and other confounding conditions (e.g. physical activity)	
	sampling material	Use sampling devices and collection tubes/containers from the same manufacturer throughout the entire study Check the absence of chemicals released from sampling devices that may interfere in subsequent metabolomics analysis (e.g. plasticizers, slip agents)	
	sampling procedure	Both serum and plasma are appropriate for blood metabolomics, with minor differences between them For serum, the use of non-coated blood collection tubes is recommended For plasma, various anticoagulants can be employed with complementary metabolomics coverage. Citrate tubes should be avoided because hamper the extraction of lipids and make impossible the determination of citric acid and related metabolites Use the same extraction site (venous vs. artery blood) throughout the entire study	Collection of spot, timed or 24-h samples depending on the study aims For spot urine samples, mid-stream collection is preferred to minimize contamination For spot urine samples, first morning collection provides the highest sensitivity, but also higher influence of diet For spot urine samples, the implementation of a normalization strategy is mandatory for correcting inter-sample variability due to dilution factors For timed and 24-h sampling, individual urine samples must be maintained under refrigeration until pooling
sample pre-processing	centrifugation and/or filtration	Incubate blood collection tubes for 30 min at RT for obtaining serum (not needed for plasma) Centrifuge blood tubes at 4 °C under reproducible conditions to separate serum/plasma from the cellular fraction (e.g. 2000 g for 10 min at 4 °C) Minimize the time delay and control the temperature between collection and further processing: < 30 min at RT, 1–2 h at 4 °C Measure metabolite ratios to determine the quality of blood samples (e.g. ascorbate/lactate, lactate/glucose, ornithine/arginine, LPC/PC) Discard hemolyzed samples	Mild pre-centrifugation (e.g. 2000 g for 10 min at 4 °C) is normally preferred over filtration to avoid adsorptive losses of metabolites Minimize the time delay and control the temperature between collection and further processing: up to 24 h at 4 °C
	preservatives	Not advised	Avoid the use of preservatives unless urine samples cannot be immediately pre-processed and stored at cold temperatures (e.g. metabolic cages). In that latter case, use sodium azide instead of boric acid
	quenching	Snap-freeze in liquid nitrogen (not dry ice)	
sample aliquoting		Aliquot samples at the moment of sampling and before freezing Prepare enough number and volume of aliquots to fulfill the analytical requirements (e.g. 50–100 μL per aliquot) Use tubes from the same manufacturer throughout the entire study	
sample transport		Samples must be transported after pre-processing, aliquoting and freezing, whenever possible Shipment at cold temperatures to avoid freeze-thaw cycles and metabolic conversions	
sample storage	short-term storage	Avoid whenever possible, apply only if ultra-freezers are not available in the collection site −20 °C for up to 1 week 4 °C for up to 1–2 h	Avoid whenever possible, apply only if ultra-freezers are not available in the collection site −20 °C for up to 6 months 4 °C for up to 24–48 h
	long-term storage	Ultra-freezer (−80 °C) for up to 5 years	
sample thawing		Stepwise thawing at 4 °C Maximum of 2–3 freeze-thaw cycles Analyze samples that have suffered the same number of freeze-thaw cycles	
